# Research Advances in AP2/ERF Transcription Factors in Rice Growth and Development

**DOI:** 10.3390/plants14172673

**Published:** 2025-08-27

**Authors:** Ying He, Ruiqi Li, Dike Li, Xingyi Fan, Xiaoyuan Chen, Chuxiong Zhuang, Jing Li

**Affiliations:** 1 State Key Laboratory for Conservation and Utilization of Subtropical Agro-Bioresources, College of Life Sciences, South China Agricultural University, Guangzhou 510642, China; 15876596509@163.com (Y.H.); lrq0821@163.com (R.L.); dk_maro@163.com (D.L.); fanwangyi961103@163.com (X.F.); 2 Guangdong Provincial Key Laboratory of Utilization and Conservation of Food and Medicinal Resources in Northern Region, Shaoguan University, Shaoguan 512005, China; chenxy2@163.com

**Keywords:** AP2/ERF transcription factors, rice, growth and development, molecular mechanisms, breeding applications

## Abstract

The AP2/ERF transcription factor family plays a vital role in regulating rice growth and development. Recent years have seen notable progress in understanding the functions of AP2/ERF transcription factors in rice. Studies indicate that these factors not only control the differentiation of rice inflorescence meristems but also participate in developing organs such as roots, stems, and leaves. However, the specific molecular mechanisms of AP2/ERF transcription factors, their interactions with other proteins, and how they precisely regulate the expression of particular genes still require further research. This paper systematically reviews recent advances in the functional studies of AP2/ERF transcription factors in rice growth and development, focusing on their roles in inflorescence development, grain formation, and the development of roots, stems, and leaves. It also discusses their potential applications in molecular breeding. By compiling recent research findings, this review aims to provide both theoretical insights and practical guidance for a better understanding of the regulatory networks involving AP2/ERF transcription factors and their use in rice genetic improvement.

## 1. Introduction

The AP2/ERF (APETALA2/ethylene-responsive element-binding factor) transcription factor family is a highly conserved regulatory protein family in plants, first isolated and named from *Arabidopsis thaliana* by Jofuku et al. [1]. Members of this family regulate the spatiotemporal expression patterns of downstream target genes by binding to specific cis-acting elements such as GCC-boxes, thereby comprehensively regulating plant growth and development. Studies have shown that AP2/ERF transcription factors play critical roles in key developmental processes, including embryonic morphogenesis [2,3], root system development [4,5,6,7], floral organ differentiation [8,9,10,11], and seed germination regulation [12,13,14].

As one of the world’s most important food crops, the yield and quality of rice (*Oryza sativa* L.) are constrained by multiple biotic and abiotic stress factors. AP2/ERF transcription factors directly participate in the regulation of rice panicle architecture formation, grain plumpness, and yield formation by precisely regulating key processes such as inflorescence meristem activity, cell division, and differentiation [15,16,17,18,19]. Meanwhile, AP2/ERF transcription factors play a core role in the molecular networks governing rice growth, development, and stress responses, with their upstream/downstream regulatory mechanisms and interactions with other signaling pathways being key focuses of current research. With the advancement of molecular biology, precise modification of AP2/ERF transcription factors via biotechnologies such as gene editing can effectively improve rice plant architecture, enhance yield potential, and strengthen stress resistance, providing important theoretical foundations and technical support for the molecular design breeding of breakthrough rice varieties.

## 2. Structural Characteristics of AP2/ERF Transcription Factors

Transcription factors (TFs) are a class of proteins that specifically bind to cis-acting elements in the promoter regions of genes and regulate the transcription of target genes through activation or repression mechanisms [20]. A typical transcription factor contains four functional domains: a DNA-binding domain (which directly recognizes cis-acting elements), a transcriptional regulation domain (which recruits co-activators or co-repressors to regulate transcription efficiency), a nuclear localization signal domain (which directs the protein into the nucleus), and an oligomerization site (which mediates interactions between homologous or heterologous proteins) [21]. In higher plants, approximately 60 transcription factor families have been identified, including AP2/ERF, ARF, bHLH, bZIP, C2H2, Dof, HSF, MYB, NAC, and WRKY. These families participate in plant growth and development, metabolic regulation, and stress responses by constructing complex regulatory networks [22].

The AP2/ERF transcription factor family is a group of plant-specific regulatory factors, named after the discovery of the APETALA2 (AP2) gene in *Arabidopsis thaliana* (involved in flower organ development) and the ethylene-response element binding factor (ERF) in tobacco [1,23,24]. All members of this family contain a highly conserved AP2/ERF domain, composed of 60–70 amino acid residues, with a three-dimensional structure consisting of one α-helix and three β-sheets [25]. This domain can be further divided into two functional modules: the YRG element (19–22 amino acids, a basic hydrophilic region responsible for recognizing cis-acting elements in promoters) and the RAYD element (42–43 amino acids, forming an amphipathic α-helix that may participate in protein interactions) [26].

**Figure 1:**
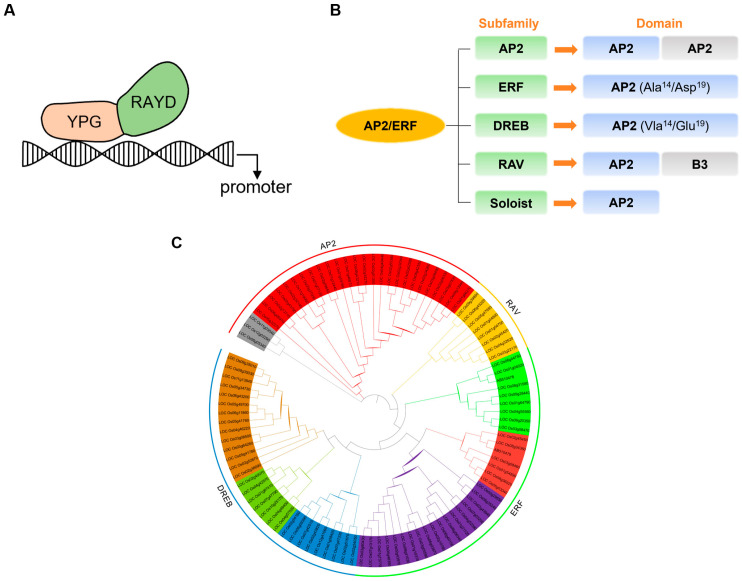
Analysis of the rice AP2/ERF transcription factor family. (**A**) The conserved domain of AP2/ERF transcription factors. The conserved domain of AP2/ERF is divided into two functional modules: the YRG element is a basic hydrophilic region responsible for recognizing cis-acting elements in promoters) and the RAYD element, that may participate in protein interactions. (**B**) The AP2/ERF family is classified into five subfamilies: the AP2 subfamily (characterized by two tandem AP2 domains); the ERF subfamily (containing a single AP2 domain with Ala^14^ and Asp^19^); the DREB subfamily (containing a single AP2 domain with Val^14^ and Glu^19^); the RAV subfamily (containing both an AP2 domain and a B3 domain); and the Soloist subfamily (containing a single, highly divergent AP2-like domain). (**C**): Phylogenetic analysis of *OsAP2/ERFs*. The different colors indicate different groups of the OsAP2/ERF family. The red color shows the AP2 group of genes, the orange color indicates the RAV group of genes, the green color shows the ERF group, and the blue color shows DREB family factors.

Based on the number and sequence characteristics of AP2 domains, the AP2/ERF family is divided into five subfamilies: AP2 (containing two tandem AP2 domains), ERF (single AP2 domain with Ala/Asp at positions 14/19), DREB (single AP2 domain with Val/Glu at positions 14/19), RAV (containing both AP2 and B3 domains), and Soloist (single highly divergent AP2 domain) [27] (Figure 1). Although all five subfamilies contain at least one AP2/ERF domain, they exhibit significant differences in other amino acid sequences, which may be related to their diverse biological functions. Among them, the ERF and DREB subfamilies specifically bind to GCC-box and DRE/CRT cis-acting elements, respectively, due to differences in the 14th/19th amino acid residues (Ala/Asp vs. Val/Glu), thereby regulating genes responsive to biotic stresses (e.g., pathogen infection) and abiotic stresses (e.g., drought, high salinity) [28,29]. Members of the AP2 subfamily (e.g., AP2, ANT) primarily participate in plant developmental processes, including inflorescence meristem formation, floral organ development, and seed formation [30,31,32]. The RAV subfamily integrates the functions of AP2 and B3 domains to regulate photoperiod and leaf senescence [33,34]. The Soloist subfamily, characterized by its unique structure and limited research, has an unclear function to date. Comparative species studies have shown that the AP2/ERF family in *Arabidopsis thaliana* contains 18 AP2, 65 ERF, 57 DREB, 6 RAV, and 1 Soloist members, while in rice, there are 26 AP2, 79 ERF, 52 DREB, 7 RAV members, with no Soloist subfamily identified [9].

## 3. Research Progress on AP2/ERF Regulate Rice Growth and Development

AP2/ERF transcription factors, as key regulatory factors in plant growth, development, and stress responses, are widely present in multiple plant species such as *Arabidopsis thaliana*, rice, soybean, maize, grape, wheat, barley, apple, and eggplant [34,35,36,37]. Although functional studies on members of this family in the model plant *Arabidopsis thaliana* have been relatively systematic, the important roles of AP2/ERF transcription factors in rice growth and development are attracting increasing attention as research progresses. Studies have shown that members of the AP2/ERF family participate in multiple rice growth and development processes through complex regulatory networks, including but not limited to panicle meristem differentiation, root development, stem elongation, leaf morphogenesis, and seed development. Notably, members of this family play irreplaceable roles in critical developmental stages such as flowering time regulation and the transition from spikelet meristem to floral meristem.

### 3.1. Development of Floral Organs in Rice

**Figure 2:**
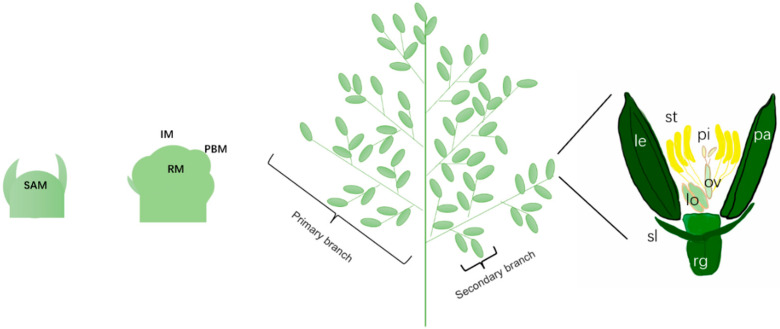
Development process of rice floral organs. The development of rice inflorescence begins with the transition from shoot apical meristem (SAM) to inflorescence meristem (IM), followed by the formation of the inflorescence axis meristem, primary stem meristem, secondary stem meristem, and, finally, the spikelet meristem (SM) and floral meristem (FM). SM will eventually differentiate into two glumes, two sterile lemmas (sl), and one flower. FM, on the other hand, will further differentiate and form the outer non reproductive organs, including 1 lemma (le), 1 palea (pa), and 2 serosa, as well as the central reproductive organs (rg), including 6 stamens (st) and 1 pistil (pi).

Floral organ development is crucial for rice, as it determines the final number of flowers and seeds formed, thereby directly affecting yield [38]. The development of rice floral organs involves a series of complex processes of meristem transformation and fate determination [39,40] (Figure 2).

The development of rice floral organs is precisely regulated by multiple transcription factors, which coordinate through complex regulatory networks to control the formation, differentiation, and morphogenesis of floral organs. Studies have shown that genes of the AP2/ERF family play a crucial role in these processes. Among them, *FZP* (*Frizzy Panicle*), a spikelet identity-determining gene, encodes a transcription factor containing an AP2/ERF domain. This gene regulates the number of spikelets by inhibiting axillary bud formation and delays the transition from spikelet meristem to floral meristem [41]. Aberrant *FZP* expression alters the number of panicle branches, consequently affecting rice yield and quality. *FZP* not only controls the transition from panicle branches to spikelets but also determines the characteristic features of floral organs by regulating the expression of MADS-box family genes [42]. To date, 21 *FZP* alleles have been identified in rice [43]. Notably, *qSBN7*, a subtype allele of *FZP*, was introduced into rice varieties via a marker-assisted backcrossing strategy by Wang et al. [44]. This significantly increased the number of secondary branches and grains per panicle; although it slightly reduced single grain length, it ultimately elevated rice yield by 10.9%. These findings underscore the significant application value of *FZP* subtype alleles in the breeding of high-quality rice varieties.

SNB (SUPERNUMERARY BRACT) and OsIDS1 (INDETERMINATE SPIKELET 1), as key transcription factors in the AP2/ERF family, play central regulatory roles in rice floral organ development. SNB primarily promotes the fate transition of spikelet meristem to floral meristem; its mutation leads to the formation of extra bracts at the base of florets, increased undeveloped glumes, ectopic lemma/palea structures and lodicules, and reduced stamen numbers [45]. Similar to SNB, OsIDS1 also plays a critical role in floral organ development. The expression pattern of *OsIDS1* is extremely similar to that of *SNB*, and its mutation results in abnormal phenotypes, such as a lemma being replaced by a supernumerary glume, elongated lodicules, and formation of extra lodicules [40]. Notably, SNB and OsIDS1 exhibit synergistic effects during inflorescence development. The double mutant *osids1/snb* displays more severe developmental defects, including premature termination or transformation of the inflorescence meristem and a significant increase in the number of bracts [46]. Genetic analysis indicates that *OsIDS1* partially compensates for the loss of *SNB* function, suggesting functional redundancy between these genes. Additionally, the regulation of *SNB* and *OsIDS1* is closely associated with miR172. Overexpression of miR172 produces a phenotype similar to the *SNB* mutation [47,48], indicating that these three components form a fine-tuned regulatory network coordinating the development of rice inflorescence and floral organs.

**Figure 3:**
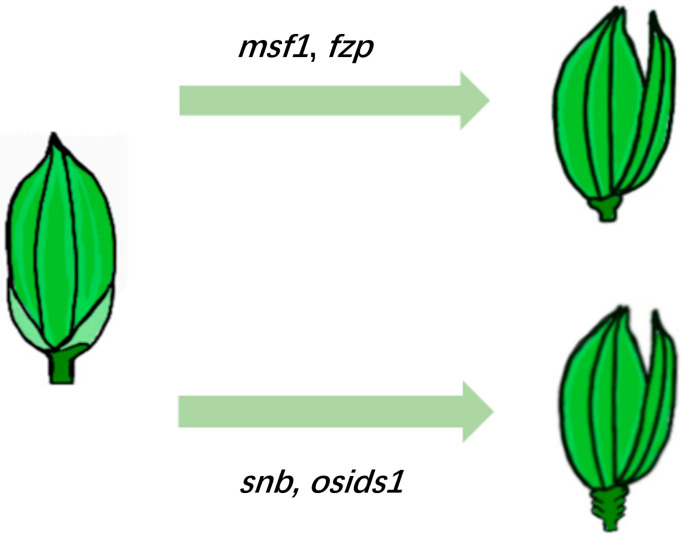
Regulation of rice floral organ development by AP2/ERF transcription factors. MFS1 promotes the expression of IDS1-type genes (e.g., *SNB*, *OsIDS1*) and spikelet development genes (e.g., *G1*/*LONG STERILE LEMMA*), thereby regulating the maintenance of spikelet meristem determinacy and organ identity specification.

MFS1 (MULTI-FLORET SPIKELET1), a subfamily member of the ERF transcription factor family, plays a key regulatory role in the establishment of the spikelet meristem (SM) and the formation of floral organ characteristics. Studies have shown that the distinct developmental defects of the *mfs1* mutant are characterized by delayed transition of SM to FM, accompanied by the formation of extra lemma-like organs within the spikelet, abnormal elongation of the spikelet axis, and degeneration of the lemma and palea, as well as transformation of the lemma into a supernumerary glume [49]. Molecular mechanism investigations reveal that MFS1 positively regulates the expression of *IDS1*-like genes (e.g., *SNB* and *OsIDS1*) and spikelet development-related genes (e.g., *G1* [*LONG STERILE LEMMA*]), thereby participating in the regulation of spikelet meristem determinacy maintenance and organ characteristic formation [50,51,52] (Figure 3). These findings indicate that *MFS1* is critical for the temporal regulation of the transition from spikelet meristem to floral meristem.

Rice flowering time, a key agronomic trait affecting yield and quality formation, is regulated by complex genetic networks. Studies have shown that AP2/ERF transcription factors OsIDS1 and SNB play critical roles in regulation of rice flowering time. Lee et al. found that overexpression of *OsIDS1* and *SNB* significantly delays rice flowering time, and this delaying effect becomes more pronounced when they are co-overexpressed with miR172 [48]. Additionally, *LFS* (*LATE FLOWERING SEMI-DWARF*), another AP2/ERF transcription factor, promotes flowering under long-day conditions. Its expression exhibits diurnal rhythmicity, peaking at dawn and continuously increasing before heading. Studies have found that the *lfs* mutant exhibits delayed flowering under long-day conditions but shows no significant phenotypic changes under short-day conditions. Molecular mechanism investigations reveal that *LFS* directly binds to the promoter of *OsLFL1* (*LEAFY COTYLEDON2* and *FUSCA3-LIKE 1*) to inhibit its expression [53]. Future research should focus on elucidating the regulatory networks of AP2/ERF transcription factors and their interaction mechanisms with other flowering-related factors, providing a theoretical basis for breeding new early-maturing and high-yielding rice varieties.

In summary, the AP2/ERF transcription factor family critically regulates floral organ development and flowering time in rice (Table 1). By precisely regulating meristem determinacy, it ensures the normal development of floral organs and the timely transition to flowering. During the development of rice floral organs, *FZP* is capable of regulating the number of spikelets. Meanwhile, *SNB* and *OsIDS1* synergistically control the inflorescence structure and the establishment of floral meristems. Finally, *LFS* is involved in the regulation of flowering time. In depth studies on AP2/ERF transcription factors and their regulatory networks not only help elucidate the molecular mechanisms underlying floral organ development and flowering time control in rice but also provide essential theoretical foundations and innovative strategies for improving rice yield and related agronomic traits.

### 3.2. AP2/ERF Related to Seed Development

The developmental process of rice seeds is precisely regulated by a multi-level transcriptional regulatory network, in which the AP2/ERF transcription factor family, as a key regulatory component, dynamically modulates the spatiotemporal expression patterns of downstream target genes, thereby playing a critical role in seed development.

Multiple AP2/ERF family transcription factors in rice have been confirmed to regulate grain development. *OsLG3* (*Oryza sativa LEAFY COTYLEDON3*), a member of this family, encodes a phosphatase that modulates the brassinosteroid (BR) signaling pathway, thereby positively regulating grain length. Studies have found that *OsLG3* not only significantly increases rice grain length and yield but also maintains grain quality [53]. Another ERF family member, *OsERF115*, is specifically expressed in rice grains and exerts a positive regulatory effect on grain size and weight. Overexpression of *OsERF115* promotes longitudinal cell elongation of the lemma and transverse cell division, enhances endosperm filling activity, and thus significantly increases grain length, width, thickness, and weight. Conversely, the *OsERF115* knockout mutant exhibits significantly reduced grain width and 1000-grain weight [16,26]. Further research has revealed that increasing *OsERF115* expression also promotes proliferation of endosperm aleurone layer cells and accelerates grain filling, ultimately leading to increased grain weight [16].

Unlike the above-mentioned positive regulators, *OsSNB*, a regulator of the brassinosteroid and auxin signaling pathways, negatively controls grain size. Jiang et al. [54] found that the *OsSNB* knockout mutant shows increased grain length, width, and weight, while overexpressing plants display the opposite phenotype. Notably, the *OsSNB* mutant allele *ssh1* generates a genetic effect of increased grain length and weight due to altered mRNA splicing patterns. Additionally, *OsSNB* influences grain shape by regulating the transcriptional levels of key genes such as *GS5* and *TGW6* [54]. Furthermore, the *FZP* gene has also been confirmed to possess multiple regulatory functions. This gene not only participates in spikelet determinacy regulation but also positively regulates seed size [42]. Ren et al. (2018) reported that the weak *fzp-12* mutant leads to reduced seed size accompanied by degeneration of sterile lemmas [55].

SERF1 (SALT-RESPONSIVE ERF1) is a key ERF transcription factor in rice, which not only responds to salt stress but also participates in regulating seed filling. Studies have shown that SERF1 influences grain development by directly regulating RPBF (RICE PROLAMIN-BOX BINDING FACTOR). Loss of function of SERF1 upregulates RPBF expression, promotes grain enlargement, and upregulates the expression of starch synthesis-related genes. Conversely, overexpression of SERF1 inhibits RPBF expression, leading to reduced grain size [56]. Additionally, RSR1 (Rice Starch Regulator1), a transcription factor of the AP2/EREBP family, affects seed size by negatively regulating the expression of starch synthesis genes. In the *rsr1* mutant, enhanced expression of starch synthesis genes, increased amylose content, and significantly enlarged seed volume and yield are observed. Notably, the loss of function of RSR1 also elevates abscisic acid (ABA) content, thereby improving rice grain quality under high temperature stress [19].

AP2 transcription factors play a critical regulatory role in rice embryonic development. Among these, BBM1 (BABY BOOM1) is a key inducer of early embryonic development. Its ectopic expression can induce the formation of somatic embryos from fertilized eggs and even trigger parthenogenesis of the egg cells [57]. Genetic analysis indicates that BBM2 and BBM3 may exhibit functional redundancy, as the *bbm1/bbm2/bbm3* triple mutant constructed using CRISPR-Cas9 technology exhibits arrested embryonic development or defects in organ differentiation, leading to significantly reduced seed viability [58]. Additionally, OsERF115 forms a protein complex with OsNF-YB1 (NUCLEAR FACTOR Y) to specifically regulate the transcription of endosperm development-related genes, thereby influencing the grain filling process [59].

Mutations in the *OsSNB* gene (e.g., the *ssh1* allele) inhibit the normal development of the rice abscission zone and cause vascular bundle thickening, significantly reducing seed shattering. Studies have shown that this mutation weakens the positive regulatory effect of *OsSNB* on the two shattering-related genes, *qSH1* (SEED SHATTERING 1) and *SH5* (SEED SHATTERING 5), further affecting lignin deposition in the abscission zone and the developmental process of the abscission zone; this confirms that *OsSNB* plays a critical role in regulating rice seed shattering [54]. During rice domestication, genetic variation in the *SH4* gene (substitution of lysine at position 79 with asparagine) leads to the loss of abscission zone cells in cultivated rice, significantly reducing seed shattering [60]. Both *SH4* and *SHAT1* (*SHATTERING ABORTION 1*) belong to the AP2/ERF transcription factor family and they are continuously expressed during early spikelet development to maintain the characteristics of the abscission zone [61]. Molecular mechanism studies have revealed that *SH4* enhances the expression of *SHAT1* in the abscission zone, while *SHAT1* maintains the expression of *SH4*, forming a positive feedback regulatory loop. *qSH1* is located downstream of this regulatory network, ensuring normal formation of the abscission zone by promoting the sustained expression of *SH4* and *SHAT1* [61].

Multiple AP2/ERF transcription factors are involved in rice seed development, including positive regulatory genes such as *OsLG3*, *OsERF115*, and *FZP*; negative regulatory genes such as *OsSNB*, *SERF1*, and *RSR1*; and shattering-related genes such as *SH4* and *SHAT1*. These AP2/ERF transcription factors regulate the seed development process through complex molecular mechanisms and diverse signaling pathways. These studies systematically reveal the molecular regulatory networks underlying key agronomic traits such as seed size, grain filling, and seed shattering but also provide critical molecular targets for rice genetic improvement, laying a theoretical foundation for precision molecular design breeding (Table 2). Based on current research progress, future work should focus on elucidating the regulatory mechanisms of these transcription factors under different environmental conditions. By integrating gene editing technologies to precisely modify these key genes, we can synergistically optimize rice yield, grain morphology, and quality traits, thereby promoting the sustainable development of agricultural production.

## 4. Research on AP2/ERF Transcription Factors Related to Root, Stem, and Leaf Development in Rice

### 4.1. AP2/ERF Related to Root Development

The rice root system plays a crucial role in growth and development, with its primary functions including anchoring the plant, absorbing water and nutrients, and ensuring that rice acquires essential soil nutrients. Recent studies have uncovered the critical regulatory roles of the AP2/ERF transcription factor family in root development (Table 3).

The development of rice crown roots is coordinately regulated by members of the AP2/ERF transcription factor family. Studies have shown that *Crl5* (CROWN ROOTLESS 5) is primarily expressed in the stem node region. Its expression is induced by auxin and promotes crown root formation by upregulating the *OsRR1* gene, which suppresses cytokinin signaling [62]. *Crl5* and *Crl1* collectively regulate adventitious root formation through distinct genetic pathways, with their double mutant exhibiting additive effects. Additionally, overexpression of *Crl5* leads to a cytokinin-insensitive phenotype, confirming its critical role in root formation [63]. ERF3 (Ethylene-Responsive Factor 3), a phosphorylation target of GUDK kinase, interacts with the WOX11 protein to regulate crown root development [6]. ERF3 directly binds to and positively regulates the *RR2* (Response Regulator 2) gene to control crown root initiation, whereas during the elongation stage of crown roots, the ERF3-WOX11 complex may suppress *RR2* expression. Genetic evidence demonstrates that knockout of *ERF3* causes root development defects, while its overexpression promotes crown root formation and primary root elongation. Furthermore, *OsAP2*/*ERF-40* participates in regulating the OsERF3-WOX11-RR2 pathway by dose-dependently activating auxin-responsive genes, thereby influencing the development of adventitious root primordia [64]. These findings reveal that AP2/ERF family members integrate auxin and cytokinin signaling to finely regulate the molecular network underlying rice root system establishment across different developmental stages.

*OsERF71* (Ethylene-Responsive Factor 71) is primarily expressed in root meristems, pericycle, and endodermis. Its overexpression significantly upregulates the expression of cell wall loosening-related genes and lignin synthesis genes, leading to notable structural changes in roots, including enlarged aerenchyma and elevated lignification levels, and these alterations are closely associated with enhanced root adaptability [65,66]. Additionally, *OsERF48*, also known as DROUGHT RESPONSIVE AP2/EREBP 1 (*OsDRAP1*), promotes root growth by regulating the expression of the calmodulin gene *OsCML16* (CALMODULIN 16). Studies have shown that plants overexpressing *OsERF48* exhibit significantly enhanced root growth, including longer primary roots, increased lateral roots, and greater root dry weight, whereas plants subjected to RNAi interference of *OsERF48* display a marked reduction in lateral roots [67].

The AP2/ERF transcription factors *Crl5* and *ERF3* are involved in the formation of crown roots in rice. *OsAP2/ERF-40* is specifically expressed in adventitious root primordia and regulates root development, together with *OsERF48/OsDRAP1*. In addition, *OsERF71* can modulate cell wall relaxation and lignin synthesis, thereby enhancing root adaptability. In recent years, the critical roles of AP2/ERF transcription factors in rice root development have garnered increasing attention. Further research should explore the regulatory mechanisms of these transcription factors under different environmental conditions, particularly their interactions with other signaling pathways. These findings will not only improve root system adaptability but also provide new potential strategies for enhancing rice yield and stress resistance.

### 4.2. AP2/ERF Related to Stem Development

The development of rice stems directly influences plant mechanical strength and lodging resistance, thereby affecting yield. Key processes in stem development are the thickening of secondary cell walls and lignin deposition, which are critical for supporting the weight of the panicle and maintaining the uprightness of rice plants.

*OsERF34* (*APETALA2/ETHYLENE RESPONSE FACTOR*) positively regulates secondary cell wall synthesis and mechanical strength by directly promoting the expression of the morphological determinant *RMD* (Rice Morphology Determinant). In *erf34* and *rmd-1* mutants, cellulose and lignin contents are significantly reduced and secondary cell walls become thinner, reducing internode mechanical strength. Conversely, plants overexpressing *OsERF34* exhibit thickened secondary cell walls and enhanced mechanical strength. This indicates that *OsERF34* plays a critical role in maintaining stem structural integrity and mechanical strength, which are of great significance for rice stability and lodging resistance [68,69].

Beyond secondary cell wall synthesis, plant hormone signaling pathways also participate in regulating stem development. In transgenic plants overexpressing *OsRPH1*, endogenous gibberellin (GA) content is significantly reduced, resulting in shortened plant height, internode length, and leaf sheath length. This further demonstrates the important role of *OsRPH1* in regulating stem development, with its mechanism likely closely related to the GA signaling pathway [70]. Plants of overexpressed *OsERF83* display stronger drought tolerance but this is accompanied by shortened stem and panicle lengths, reduced grain width, and decreased yield. This suggests that, while *OsERF83* has application potential in stress resistance, its adverse effects on stem development need to be balanced during breeding [71,72].

During the stem development of rice, *OsRPH1* regulates stem length and plant height through the GA pathway. *OsERF34* can positively regulate the synthesis of secondary cell walls and mechanical strength, while *OsERF83* negatively regulates stem length and simultaneously enhances rice drought resistance. Overall, rice stem development is not only influenced by secondary cell wall synthesis but also closely regulated by complex plant hormone signaling pathways (Table 4). During genetic improvement, the balance between stem development and plant stability must be comprehensively considered.

### 4.3. AP2/ERF Related to Leaf Development

Rice leaves, as the primary organs for photosynthesis, are critical for rice growth, yield, and stress tolerance. Leaf health directly influences photosynthetic efficiency, grain production, and the ability to tolerate environmental stresses.

The *SUB1A* (*SUBMERGENCE1A*) gene plays a pivotal role in regulating leaf senescence. By maintaining chlorophyll and carbohydrate reserves, *SUB1A* suppresses ethylene accumulation and mitigates the effects of jasmonic acid and salicylic acid on leaf senescence, thereby delaying leaf aging. This function enhances rice tolerance to submergence, drought, and oxidative stresses [73,74]. *OsERF101*, a key transcription factor, promotes leaf senescence by binding to the promoter regions of senescence-related genes *OsNAP* and *OsMYC2* and activating their expression [75]. *HL6* (Hairy Leaf 6) has been identified as a critical regulator of trichome (epidermal hair) development. Overexpression of *HL6* leads to excessive trichome formation, which is closely associated with elevated indole-3-acetic acid (IAA) levels. Conversely, *hl6* mutants exhibit reduced trichome density, confirming the central role of *HL6* in trichome morphogenesis [76,77].

These studies have uncovered key genes and their regulatory mechanisms underlying leaf development, providing valuable insights into rice leaf biology and adaptability (Table 5). Future research should focus on leveraging these genes to improve the photosynthetic efficiency and environmental resilience of rice through genetic improvement.

## 5. Future Prospects

As key regulators of rice growth and development, AP2/ERF transcription factors have seen significant advances in research. Researchers have not only successfully identified multiple members of this family but also initially revealed their critical regulatory roles in rice growth and development. However, numerous scientific questions remain to be addressed. Firstly, the molecular mechanisms underlying the interactions between AP2/ERF transcription factors and other proteins are incompletely elucidated. Secondly, the precision and specificity with which they regulate downstream target gene expression require further investigation. Additionally, the signal transduction pathways through which these transcription factors sense and respond to external environmental changes remain to be fully elucidated. Resolving these key scientific questions will contribute to comprehensively elucidating the core regulatory mechanisms of AP2/ERF transcription factors in the rice growth and development regulatory network, providing critical theoretical foundations for crop genetic improvement.

With the rapid development of gene editing technologies, precision editing of AP2/ERF family genes has become a reality. Gene editing tools represented by CRISPR/Cas9 provide powerful means to achieve specific regulation of AP2/ERF genes, enabling precise control of target gene expression while maintaining genomic integrity. This technological breakthrough has not only deepened our understanding of the functional mechanisms of AP2/ERF genes but also opened new avenues for rice molecular breeding. Through spatiotemporal-specific regulation of gene expression, researchers aim to precisely manipulate downstream gene networks, optimize rice growth and development processes, and enhance target traits, while avoiding undesirable phenotypes.

In summary, the role of AP2/ERF transcription factors in rice growth and development holds significant scientific and practical value, emerging as a critical area in rice functional genomics and molecular breeding research. This paper systematically reviews the research advances in this field, focusing on the mechanisms by which AP2/ERF transcription factors regulate panicle development, grain formation, and development of roots, stems, and leaves in rice. It also discusses the application prospects of these transcription factors in rice breeding, aiming to provide new perspectives and ideas for future studies on AP2/ERF transcription factors in rice functional gene research and variety improvement.

## Figures and Tables

**Table 1:** AP2/ERF transcription factors related to spike development.

Genes	Gene ID	Function Description	References
*FZP*	4344233	Control the number of spikelets, delay the transition from spikelets to flowers, and affect yield and quality.	[41,42,43,44]
*SNB*	4342787	Positive regulation of spikelet to flower transition affects inflorescence structure and floral meristem establishment.	[45,46,47,48]
*OsIDS1*	4334582	Collaborate with SNB to control inflorescence structure and floral meristem, affecting bract formation.	[47,48]
*MFS1*	4339208	Participate in the establishment of spikelet meristematic tissue and the formation of floral organ characteristics, regulating the transition time from spikelet to flower.	[49,50]
*LFS*	4345697	Promote flowering under long day conditions by inhibiting *OsLFL1* expression.	[53]

**Table 2:** AP2/ERF transcription factors related to seed development.

Genes	Gene ID	Function Description	References
*OsLG3*	4331845	Positive regulation of rice grain length which regulates BR signal transduction by encoding phosphatase, increasing grain length and yield.	[53]
*OsERF115*	4346073	Positively regulates grain size and weight, promoting elongation and division of glume cells, and increasing grain weight.	[16]
*OsSNB*	4342787	Participates in the regulation of brassinosteroid and auxin signaling, negatively regulate grain size, and its mutants increase grain length and weight.	[54]
*FZP*	4344233	Regulates the determinacy of spikelet and positively regulating seed size.	[42,55]
*SERF1*	107277887	Responds to salt stress, regulating *RPBF* expression, affecting grain filling and starch biosynthesis.	[56]
*RSR1*	4337654	Negatively regulates starch synthesis gene expression in seeds, affecting seed size and increasing amylose content.	[19]
*BBM1*, *BBM2*, *BBM3*	4350315	Plays a critical role in early embryonic development and is an inducer of embryonic development.	[57,58]
*OsSNB* (*ssh1* allele)	4342787	Regulates *qSH1* and *SH5*, affecting the deposition of lignin in the detachment zone and the normal development of the detachment layer, and reducing grain size.	[54]
*SH4*	9266435	Key transcription factors that control seed drop and affect the formation of detached cells.	[60,61]
*SHAT1*	9269072	Participates in delamination development and is crucial for genetic regulation of rice seed drop.	[61]

**Table 3:** AP2/ERF transcription factors related to root development.

Genes	Gene ID	Function Description	References
*Crl5*	4342308	Induced by auxin, upregulation of *OsRR1* inhibits cytokinin signaling and promotes crown root formation.	[62,63]
*ERF3*	4331843	Interacts with WOX11 to regulate coronal root development, controlling coronal root initiation through RR2.	[6,64]
*OsAP2/ERF-40*	4324418	Specifically expressed in adventitious root primordia, it affects root development by regulating the OsERF3-WOX11-RR2 pathway.	[64]
*OsERF71*	4340383	Expressed in root meristem tissue, affecting cell wall relaxation and lignin synthesis, enhancing root adaptability.	[65,66]
*OsERF48*/ *OsDRAP1*	4345541	Regulates *OsCML16* expression to promote root growth, including primary and lateral roots.	[67]

**Table 4:** AP2/ERF transcription factors related to stem development.

Genes	Gene ID	Function Description	References
*OsERF34*	9266374	Promotes the expression of RMD, positively regulates secondary cell wall synthesis and mechanical strength.	[68,69]
*OsRPH1*	4339670	Regulates stem development and is closely related to the gibberellin signaling pathway.	[70]
*OsERF83*	107276031	Overexpression enhances drought resistance but affects stem and spike length, leading to a decrease in yield.	[71,72]

**Table 5:** AP2/ERF transcription factors related to leaf development.

Genes	Gene ID	Function Description	References
*SUB1A*	4352338	Inhibits ethylene accumulation, weakens the effects of jasmonic acid and salicylic acid on leaf senescence, and enhances stress tolerance.	[73,74]
*OsERF101*	4335707	Combines *OsNAP* and *OsMYC2* promoters to promote leaf senescence.	[75]
*HL6*	4341722	Affects the distribution and morphology of trichomes on rice leaves and other epidermal parts.	[76,77]

## Data Availability

Not applicable.

## Abbreviations

The following abbreviations are used in this manuscript:

ABA	Abscisic acid
AP2	APETALA 2
BBM1	Baby boom 1
BR	Brassinosteroid
Crl5	Crown rootless 5
DRAP1	Drought responsive AP2/EREBP 1
ERF	Ethylene-response factor
FM	Floral meristem
FZP	Frizzy panicle
GA	Gibberellin
HL6	Hairy leaf 6
IAA	Indole-3-acetic acid
IM	Inflorescence meristem
LFS	Late flowering semi-dwarf
MFS1	Multi-floret spikelet1
OsIDS1	*Oryza sativa* indeterminate spikelet 1
OsLG3	*Oryza sativa* leafy cotyledon 3
qSH1	Seed shattering 1
RMD	Rice morphology determinant
RSR1	Rice starch regulator 1
SAM	Shoot apical meristem
SERF1	Salt-responsive ERF 1
SH5	Seed shattering 5
SHAT1	Shattering abortion 1
SM	Spikelet meristem
SNB	Supernumerary bract
SUB1A	Submergence 1A

## References

[1] Jofuku K.D., den Boer B.G., Van Montagu M., Okamuro J.K. (1994). Control of *Arabidopsis* flower and seed development by the homeotic gene *apetala2*. Plant Cell.

[2] Auman H.J., Nottoli T., Lakiza O., Winger Q., Donaldson S., Williams T. (2002). Transcription factor AP2 gamma is essential in the extraembryonic lineages for early postimplantation development. Development.

[3] Tsuwamoto R., Yokoi S., Takahata Y. (2010). *Arabidopsis* embryomaker encoding an AP2 domain transcription factor plays a key role in developmental change from vegetative to embryonic phase. Plant Mol. Biol..

[4] Lv B., Wei K., Hu K., Tian T., Zhang F., Yu Z., Zhang D., Su Y., Sang Y., Zhang X. (2021). Mpk14-mediated auxin signaling controls lateral root development via *ERF13* regulated very long chain fatty acid biosynthesis. Mol. Plant.

[5] Guyomarc’H S., Boutte Y., Laplaze L. (2021). AP2/ERF transcription factors orchestrate very long chain fatty acid biosynthesis during *Arabidopsis* lateral root development. Mol. Plant.

[6] Zhao Y., Cheng S., Song Y., Huang Y., Zhou S., Liu X., Zhou D.X. (2015). The interaction between rice ERF3 and WOX11 promotes crown root development by regulating gene expression involved in cytokinin signaling. Plant Cell.

[7] Kang N.Y., Lee H.W., Kim J. (2013). The AP2/EREBP gene *PUCHI* co-acts with lBD16/ASL18 and IBD18/ASL20 downstream of *ARF7* and *ARF19* to regulate lateral root development in Arabidopsis. Plant Cell Physiol..

[8] Wang Z., Song G., Zhang F., Shu X., Wang N. (2023). Functional characterization of AP2/ERF transcription factors during flower development and anthocyanin biosynthesis related candidate genes in *Lycoris*. Int. J. Mol. Sci..

[9] Jiang Q., Wang Z., Hu G., Yao X. (2022). Genome-wide identification and characterization of *AP2/ERF* gene superfamily during flower development in *Actinidia eriantha*. BMC Genom..

[10] Nakano T., Fujisawa M., Shima Y., Ito Y. (2014). The AP2/ERF transcription factor slERF52 functions in flower pedicel abscission in tomato. J. Exp. Bot..

[11] Wollmann H., Mica E., Todesco M., Long J.A., Weigel D. (2010). On reconciling the interactions between *APETALA2*, miR172 and *AGAMOUS* with the ABC model of flower development. Development.

[12] Meng H., Chen Y., Li T., Shi H., Yu S., Gao Y., Wang Z., Wang X., Zhu J.K., Hong Y. (2023). APETALA2 is involved in ABA signaling during seed germination. Plant Mol. Biol..

[13] Li Z., Sheerin D.J., von Roepenack-Lahaye E., Stahl M., Hiltbrunner A. (2022). The phytochrome interacting proteins ERF55 and ERF58 repress light-induced seed germination in *Arabidopsis thaliana*. Nat. Commun..

[14] Wang C., Wang H., Zhang J., Chen S. (2008). A seed-specific AP2-domain transcription factor from soybean plays a certain role in regulation of seed germination. Sci. China C Life Sci..

[15] Teng K., Zhao N., Xie Y., Li R., Li J. (2024). An AP2/ERF transcription factor controls generation of the twin-seedling rice. J. Adv. Res..

[16] Liu C., Ma T., Yuan D., Zhou Y., Long Y., Li Z., Dong Z., Duan M., Yu D., Jing Y. (2022). The *OsEIL1*-*OsERF115*-target gene regulatory module controls grain size and weight in rice. Plant Biotechnol. J..

[17] Harrop T., Mantegazza O., Luong A.M., Bethune K., Lorieux M., Jouannic S., Adam H. (2019). A set of AP2-like genes is associated with inflorescence branching and architecture in domesticated rice. J. Exp. Bot..

[18] Qi W., Sun F., Wang Q., Chen M., Huang Y., Feng Y.Q., Luo X., Yang J. (2011). Rice ethylene-response AP2/ERF factor *OsEATB* restricts internode elongation by down-regulating a gibberellin biosynthetic gene. Plant Physiol..

[19] Fu F.F., Xue H.W. (2010). Coexpression analysis identifies rice starch regulator1, a rice AP2/EREBP family transcription factor, as a novel rice starch biosynthesis regulator. Plant Physiol..

[20] Liu W., Stewart C.J. (2016). Plant synthetic promoters and transcription factors. Curr. Opin. Biotechnol..

[21] Franco-Zorrilla J.M., Solano R. (2017). Identification of plant transcription factor target sequences. Biochim. Biophys. Acta Gene Regul. Mech..

[22] Feng K., Hou X.L., Xing G.M., Liu J.X., Duan A.Q., Xu Z.S., Li M.Y., Zhuang J., Xiong A.S. (2020). Advances in AP2/ERF super-family transcription factors in plant. Crit. Rev. Biotechnol..

[23] Shinshi H., Usami S., Ohme-Takagi M. (1995). Identification of an ethylene-responsive region in the promoter of a tobacco class I chitinase gene. Plant Mol. Biol..

[24] Ohme-Takagi M., Shinshi H. (1995). Ethylene-inducible DNA binding proteins that interact with an ethylene-responsive element. Plant Cell.

[25] Nakano T., Suzuki K., Fujimura T., Shinshi H. (2006). Genome-wide analysis of the ERF gene family in *Arabidopsis* and rice. Plant Physiol..

[26] Weigel D. (1995). The APETALA2 domain is related to a novel type of DNA binding domain. Plant Cell.

[27] Licausi F., Ohme-Takagi M., Perata P. (2013). Apetala2/ethylene responsive factor (AP2/ERF) transcription factors: Mediators of tress responses and developmental programs. New Phytol..

[28] Xu L., Yang L., Li A., Guo J., Wang H., Qi H., Li M., Yang P., Song S. (2024). An AP2/ERF transcription factor confers chilling tolerance in rice. Sci. Adv..

[29] Xie Z., Nolan T.M., Jiang H., Yin Y. (2019). AP2/ERF transcription factor regulatory networks in hormone and abiotic stress responses in Arabidopsis. Front. Plant Sci..

[30] Jofuku K.D., Omidyar P.K., Gee Z., Okamuro J.K. (2005). Control of seed mass and seed yield by the floral homeotic gene *APETALA2*. Proc. Natl. Acad. Sci. USA.

[31] Maes T., Van de Steene N., Zethof J., Karimi M., D’Hauw M., Mares G., Van Montagu M., Gerats T. (2001). *PETUNIA* AP2-like genes and their role in flower and seed development. Plant Cell.

[32] Zong Y., Hao Z., Tu Z., Shen Y., Zhang C., Wen S., Yang L., Ma J., Li H. (2021). Genome-wide survey and identification of AP2/ERF genes involved in shoot and leaf development in liriodendron chinense. BMC Genom..

[33] Woo H.R., Kim J.H., Kim J., Kim J., Lee U., Song I.J., Kim J.H., Lee H.Y., Nam H.G., Lim P.O. (2010). The rav1 transcription factor positively regulates leaf senescence in Arabidopsis. J. Exp. Bot..

[34] Zhu X., Yan X., Li W., Zhang M., Leng J., Yu Q., Liu L., Xue D., Zhang D., Ding Z. (2025). GmERF13 mediates salt inhibition of nodulation through interacting with GmLBD16a in soybean. Nat. Commun..

[35] Hu Z., Wang X., Wei L., Wansee S., Rabbani N.H., Chen L., Kang Z., Wang J. (2023). Taap2-10, an AP2/ERF transcription factor, contributes to wheat resistance against stripe rust. J. Plant Physiol..

[36] Wang M., Gao M., Zhao Y., Chen Y., Wu L., Yin H., Xiong S., Wang S., Wang J., Yang Y. (2022). LcERF19, an AP2/ERF transcription factor from *Litsea cubeba*, positively regulates geranial and neral biosynthesis. Hortic. Res..

[37] Xue G.P., Loveridge C.W. (2004). *Hvdrf1* is involved in abscisic acid-mediated gene regulation in barley and produces two forms of AP2 transcriptional activators, interacting preferably with a CT-rich element. Plant J..

[38] Ray D.K., Mueller N.D., West P.C., Foley J.A. (2013). Yield trends are insufficient to double global crop production by 2050. PLoS ONE.

[39] Zhang D., Yuan Z. (2014). Molecular control of grass inflorescence development. Annu. Rev. Plant Biol..

[40] Thompson B.E., Hake S. (2009). Translational biology: From *Arabidopsis* flowers to grass inflorescence architecture. Plant Physiol..

[41] Wang S.S., Chung C.L., Chen K.Y., Chen R.K. (2020). A novel variation in the *Frizzle Panicle* (*FZP*) gene promoter improves grain number and yield in rice. Genetics.

[42] Xing H., Wang H., Huang Y., Ma X., Wu S., Li Y., Sun C., Sun H. (2025). *FZP* modulates tillering via *OsMADS57* in rice. Plant Biotechnol. J..

[43] Wang W., Chen W., Wang J. (2023). *Frizzle Panicle* (*FZP*) regulates rice spikelets development through modulating cytokinin metabolism. BMC Plant Biol..

[44] Wang S.S., Chen R.K., Chen K.Y., Liu C.Y., Kao S.M., Chung C.L. (2017). Genetic mapping of the *qSBN7* locus, a QTL controlling secondary branch number per panicle in rice. Breed. Sci..

[45] Lee D.Y., Lee J., Moon S., Park S.Y., An G. (2007). The rice heterochronic gene *supernumerary bract* regulates the transition from spikelet meristem to floral meristem. Plant J..

[46] Lee D.Y., An G. (2012). Two AP2 family genes, *Supernumerary Bract* (*SNB*) and *OsIndeterminate Spikelet 1* (*osIDS1*), synergistically control inflorescence architecture and floral meristem establishment in rice. Plant J..

[47] Lee Y.S., Lee D.Y., Cho L.H., An G. (2014). Rice *miR172* induces flowering by suppressing *OsIDS1* and *SNB*, two AP2 genes that negatively regulate expression of *Ehd1* and florigens. Rice.

[48] Ji H., Han C.D., Lee G.S., Jung K.H., Kang D.Y., Oh J., Oh H., Cheon K.S., Kim S.L., Choi I. (2019). Mutations in the microRNA172 binding site of *Supernumerary Bract* (*SNB*) suppress internode elongation in rice. Rice.

[49] Ren D., Li Y., Zhao F., Sang X., Shi J., Wang N., Guo S., Ling Y., Zhang C., Yang Z. (2013). *Multi-Floret Spikelet1*, which encodes an AP2/ERF protein, determines spikelet meristem fate and sterile lemma identity in rice. Plant Physiol..

[50] Wu T., Ali A., Wang J., Song J., Fang Y., Zhou T., Luo Y., Zhang H., Chen X., Liao Y. (2021). A homologous gene of *OsREL2*/*ASP1*, *ASP*-*LSL* regulates pleiotropic phenotype including long sterile lemma in rice. BMC Plant Biol..

[51] Song S., Wang G., Hu Y., Liu H., Bai X., Qin R., Xing Y. (2018). OsMFT1 increases spikelets per panicle and delays heading date in rice by suppressing *EHD1*, *FZP* and *SEPALLATA*-like genes. J. Exp. Bot..

[52] Shim Y., Lim C., Seong G., Choi Y., Kang K., Paek N.C. (2022). The AP2/ERF transcription factor late flowering semi-dwarf suppresses long-day-dependent repression of flowering. Plant Cell Environ..

[53] Yu J., Xiong H., Zhu X., Zhang H., Li H., Miao J., Wang W., Tang Z., Zhang Z., Yao G. (2017). *OsLG3* contributing to rice grain length and yield was mined by ho-lamap. BMC Biol..

[54] Jiang L., Ma X., Zhao S., Tang Y., Liu F., Gu P., Fu Y., Zhu Z., Cai H., Sun C. (2019). The APETALA2-like transcription factor supernumerary bract controls rice seed shattering and seed size. Plant Cell.

[55] Ren D., Hu J., Xu Q., Cui Y., Zhang Y., Zhou T., Rao Y., Xue D., Zeng D., Zhang G. (2018). *FZP* determines grain size and sterile lemma fate in rice. J. Exp. Bot..

[56] Schmidt R., Schippers J.H., Mieulet D., Watanabe M., Hoefgen R., Guiderdoni E., Mueller-Roeber B. (2014). Salt-responsive ERF1 is a negative regulator of grain filling and gibberellin-mediated seedling establishment in rice. Mol. Plant.

[57] Khanday I., Santos-Medellin C., Sundaresan V. (2023). Somatic embryo initiation by rice *BABY BOOM1* involves activation of zygote-expressed auxin biosynthesis genes. New Phytol..

[58] Chahal L.S., Conner J.A., Ozias-Akins P. (2022). Phylogenetically distant baby boom genes from setaria italica induce parthenogenesis in rice. Front. Plant Sci..

[59] Xu J.J., Zhang X.F., Xue H.W. (2016). Rice aleurone layer specific OsNF-YB1 regulates grain filling and endosperm development by interacting with an erf transcription factor. J. Exp. Bot..

[60] Zhang Y.L., Xia Q.Y., Jiang X.Q., Hu W., Ye X.X., Huang Q.X., Yu S.B., Guo A.P., Lu B.R. (2022). Reducing seed shattering in weedy rice by editing *SH4* and *qSH1* genes: Implications in environmental biosafety and weed control through transgene mitigation. Biology.

[61] Hofmann N.R. (2012). *SHAT1*, a new player in seed shattering of rice. Plant Cell.

[62] Kitomi Y., Ito H., Hobo T., Aya K., Kitano H., Inukai Y. (2011). The auxin responsive *AP2/ERF* transcription factor *crown rootless5* is involved in crown root initiation in rice through the induction of *OsRR1*, a type-a response regulator of cytokinin signaling. Plant J..

[63] Lavarenne J., Gonin M., Guyomarc’H S., Rouster J., Champion A., Sallaud C., Laplaze L., Gantet P., Lucas M. (2019). Inference of the gene regulatory network acting downstream of crown rootless 1 in rice reveals a regulatory cascade linking genes involved in auxin signaling, crown root initiation, and root meristem specification and maintenance. Plant J..

[64] Neogy A., Singh Z., Mushahary K., Yadav S.R. (2021). Dynamic cytokinin signaling and function of auxin in cytokinin responsive domains during rice crown root development. Plant Cell Rep..

[65] Lee D.K., Yoon S., Kim Y.S., Kim J.K. (2017). Rice *OsERF71* mediated root modification affects shoot drought tolerance. Plant Signal. Behav..

[66] Lee D.K., Jung H., Jang G., Jeong J.S., Kim Y.S., Ha S.H., Do C.Y., Kim J.K. (2016). Overexpression of the *OsERF71* transcription factor alters rice root structure and drought resistance. Plant Physiol..

[67] Jung H., Chung P.J., Park S.H., Redillas M., Kim Y.S., Suh J.W., Kim J.K. (2017). Overexpression of *OsERF48* causes regulation of *OsCML16*, a calmodulin-like protein gene that enhances root growth and drought tolerance. Plant Biotechnol. J..

[68] Zhai H., Zhou C., Zhang Y., Wang Y., Wang M., Wei S., Li T. (2025). Mechanism analysis of *OsBHLH34*-*OsERF34* mediated regulation of rice resistance to sheath blight. Int. J. Mol. Sci..

[69] Zhang J., Liu Z., Sakamoto S., Mitsuda N., Ren A., Persson S., Zhang D. (2022). Ethylene response factor 34 promotes secondary cell wall thickening and strength of rice peduncles. Plant Physiol..

[70] Ma Z., Wu T., Huang K., Jin Y.M., Li Z., Chen M., Yun S., Zhang H., Yang X., Chen H. (2020). A novel AP2/ERF transcription factor, OsRPH1, negatively regulates plant height in rice. Front. Plant Sci..

[71] Jung S.E., Bang S.W., Kim S.H., Seo J.S., Yoon H.B., Kim Y.S., Kim J.K. (2021). Overexpression of *OsERF83*, a vascular tissue-specific transcription factor gene, confers drought tolerance in rice. Int. J. Mol. Sci..

[72] Tezuka D., Kawamata A., Kato H., Saburi W., Mori H., Imai R. (2019). The rice ethylene response factor *OsERF83* positively regulates disease resistance to magnaporthe oryzae. Plant Physiol. Biochem..

[73] Lin C.C., Lee W.J., Zeng C.Y., Chou M.Y., Lin T.J., Lin C.S., Ho M.C., Shih M.C. (2023). Sub1a-1 anchors a regulatory cascade for epigenetic and transcriptional controls of submergence tolerance in rice. PNAS Nexus.

[74] Fukao T., Yeung E., Bailey-Serres J. (2011). The submergence tolerance regulator sub1a mediates crosstalk between submergence and drought tolerance in rice. Plant Cell.

[75] Lim C., Kang K., Shim Y., Sakuraba Y., An G., Paek N.C. (2020). Rice ethylene response factor 101 promotes leaf senescence through jasmonic acid-mediated regulation of *OsNAP* and *OsMYC2*. Front. Plant Sci..

[76] Sun W., Gao D., Xiong Y., Tang X., Xiao X., Wang C., Yu S. (2017). Hairy leaf 6, an AP2/ERF transcription factor, interacts with OsWOX3B and regulates trichome formation in rice. Mol. Plant.

[77] Li J., Tang B., Li Y., Li C., Guo M., Chen H., Han S., Li J., Lou Q., Sun W. (2021). Rice *SPL10* positively regulates trichome development through expression of *HL6* and auxin-related genes. J. Integr. Plant Biol..

